# Pelvic Obliquity Impacts Acetabular Coverage Measurements, and the Elevated Hip Is More Frequently Symptomatic in Hip Preservation Patients

**DOI:** 10.1002/ars2.70129

**Published:** 2026-10-29

**Authors:** Benjamin T. Johnson, Daniel C. Lewis, Allan K. Metz, Ashley L. Matter, Christopher L. Peters, Lucas A. Anderson, Stephen K. Aoki

**Affiliations:** ^1^ Department of Orthopaedics University of Utah Salt Lake City Utah U.S.A.

## Abstract

**Purpose:**

To quantify the impact of femoral head height discrepancies on pelvic‐based versus ground‐based measurements for lateral center‐edge angle (LCEA) and Tönnis angle and to determine whether these discrepancies affected the clinical presentation of patients who presented to a hip preservation clinic.

**Methods:**

Consecutive patients who underwent a primary hip arthroscopy between May 2021 and August 2021 and consecutive patients who underwent a primary periacetabular osteotomy between January 2020 and December 2023 with available preoperative standing anteroposterior radiographs were included. Patients were excluded if they (1) had undergone a previous surgery in either hip prior to the primary operation or (2) were less than 18 years of age at the time of surgery. The LCEA to the sourcil edge, LCEA to the bone edge, and Tönnis angle were calculated, referencing off (1) the pelvis and (2) the horizontal to the ground. Differences between measurements were calculated in degrees, and differences in femoral head heights to approximate pelvic obliquity were calculated in millimeters. Comparisons were made via regression analysis.

**Results:**

Fifty hip arthroscopy patients and 75 periacetabular osteotomy patients were included. Regression analysis showed that LCEA to the sourcil edge was impacted by the difference in femoral head height when comparing the pelvic‐based measurement with the ground‐based measurement (*B* = 0.298; *R* ^2^ = 0.775; 95% confidence interval = 0.274‐0.322; *P* < .001). Furthermore, in patients with a femoral head height difference of 10 mm or more, 100% (8/8) of patients who underwent a periacetabular osteotomy and 75% (6/8) of patients who underwent a hip arthroscopy reported more symptoms on the more elevated hip.

**Conclusions:**

Differences in femoral head height on standing anteroposterior radiographs impact ground‐ versus pelvic‐based radiographic measurements of acetabular coverage by approximately 0.3° per millimeter of femoral head height difference and may result in functional undercoverage of the elevated hip.

**Level of Evidence:**

Level IV, retrospective case series.

## Graphical abstract

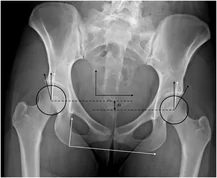

Understanding acetabular coverage is an important concept for all orthopaedic hip surgeons. Multiple methods have been used to characterize acetabular coverage including the Tönnis angle (TA) (or acetabular index), acetabular wall index, posterior wall index, anterior center‐edge angle, and lateral center‐edge angle (LCEA).^1^, ^2^, ^3^, ^4^ Although all have been used within orthopaedic literature, LCEA and TA on anteroposterior (AP) radiographs remain the most common, especially when considering a patient for hip arthroscopy or a periacetabular osteotomy (PAO).^4^ Patients are categorized as having normal coverage if their LCEA is between 25° and 40° and are categorized as under‐ or overcoverage if their LCEA is below 25° or above 40°, respectively.^4^ TA is considered normal if the measurement is between 0° and 10°.^5^ Despite the inherent limitation of these angles being a fixed 2‐dimensional measurement on a dynamic 3‐dimensional structure, this number has been used historically to guide surgical intervention of the hip.^6^, ^7^, ^8^, ^9^

Multiple methods for calculating LCEA have been proposed.^10^, ^11^, ^12^ Typically, the measurement involves drawing a best‐fit circle around the femoral head on an AP radiograph, creating a vertical line to place in the center of the circle, and determining the angle between this vertical line and either the lateral edge of the sourcil or the bony edge.^4^ There has been some debate as to whether to use the ground as the horizontal perpendicular reference for the vertical line of this angle or to reference the pelvic anatomy so as to account for pelvic obliquity.^13^, ^14^ Furthermore, within pelvic‐based measurements, debate remains as to what reference points to use including the ischia, the teardrops (such as the transtear drop line), midline of the sacrum, or the femoral heads,^13^ although differences between the measurements appear to be minimal.^10^, ^13^, ^15^

Because these measurements depend on the orientation of the pelvis, tilt and limb length discrepancies may also impact the pelvic‐based measurements.^16^ Theoretically, if a pelvis is tilted down to the right because of a shorter right leg, then the pelvic‐based measurements would show a relative acetabular overcoverage on the right and relative acetabular undercoverage on the left compared with a ground‐based measurement. Despite the impact pelvic orientation may have on pelvic‐based radiographic measurements compared with ground‐based measurements, no studies have quantified how much these measurements change with leg length discrepancies (LLDs) and whether a “functional” measurement of coverage that accounts for the impact of leg length will correlate with the individual symptomology of the patient. The purpose of this study was to quantify the impact of femoral head height discrepancies on pelvic‐based versus ground‐based measurements for LCEA and TA and to determine whether these discrepancies affected the clinical presentation of patients who presented to a hip preservation clinic. We hypothesized that there would be a substantial difference between measurement types and that the longer leg would have greater symptoms because of a decrease in functional acetabular coverage.

## METHODS

### Patient Selection

Consecutive patients who underwent a primary hip arthroscopy for femoroacetabular impingement syndrome performed by the senior author (S.K.A.) between May 2021 and August 2021 were retrospectively reviewed and included. Additionally, consecutive patients who underwent a primary PAO between January 2020 and December 2023 by 2 surgeons (C.L.P., L.A.A.) at our institution were included in the study. Patients were excluded if they had undergone a previous surgery in either hip prior to the primary operation or were less than 18 years of age at the time of surgery or did not have available preoperative standing AP radiographs. Demographic information including age, sex, and body mass index was collected.

### Surgical Indications and Symptomatic Hip Determination

Patients were indicated for hip arthroscopy if they showed radiographic (alpha angle >55.0°) and clinical evidence of femoroacetabular impingement syndrome. Patients were indicated for PAO if they showed hip pain and radiographic evidence (LCEA < 25.0) consistent with hip dysplasia. The more symptomatic hip was identified through clinical notes and as being the surgical hip. In cases of staged bilateral procedures, the more symptomatic hip was determined based on the preoperative clinical notes (lower patient‐reported outcomes) and whichever hip ended up having surgery first. This study was reviewed by the Institutional Review Board and determined to be exempt because of its retrospective nature (IRB #: 169523).

### Measurements and Data Collection

Measurements were made by a fourth‐year orthopaedic surgery resident and a fourth‐year medical student (D.C.L., B.T.J.) using Picture Archiving and Communication System. Best‐fit circles were drawn around both hips on preoperative AP radiographs. Horizontal lines were drawn across the hips from the center of the right and left femoral heads. The distance between these lines was used to determine the difference in femoral head height (LLD); however, no direct leg measurements were made (Figure 1).^17^, ^18^ Next, reference lines were drawn perpendicular to the horizontal axis of the pelvis as determined by a line below the ischia (pelvic based) and perpendicular to the ground (ground based) as determined by the radiograph cassette.^19^, ^20^ The LCEA based on the pelvis as previously described and the LCEA based on the ground to both the lateral sourcil and the lateral rim of the acetabulum were measured (Figure 2A and B).^4^ Finally, TA was sequentially measured using both the pelvis and ground as reference. For the hip arthroscopy patients, both hips were measured, giving 2 measurements for each patient. For the patients who underwent PAO, only the operative hip was measured for regression purposes.

**FIGURE 1:**
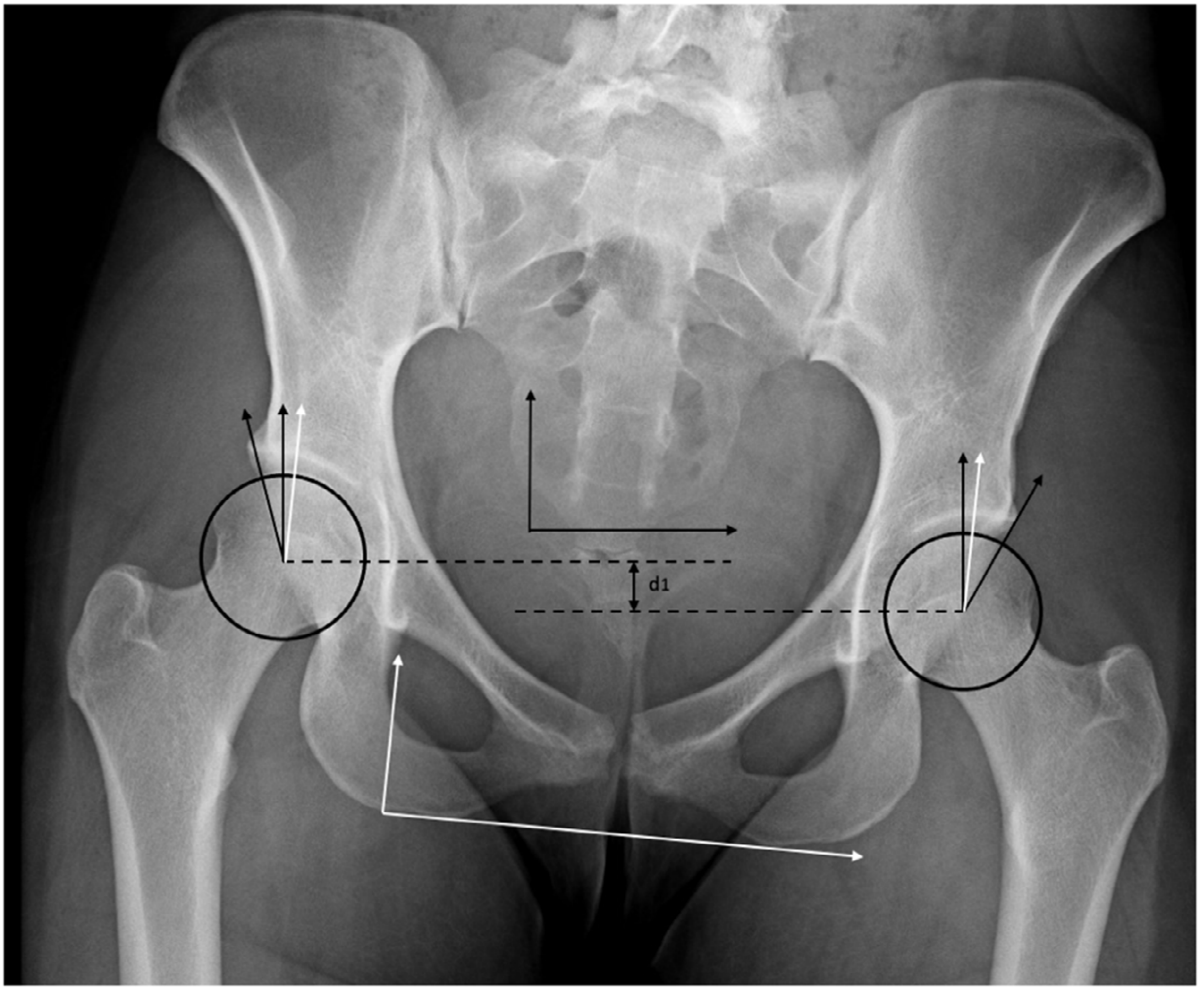
Example of an anteroposterior weight‐bearing pelvis in a patient who underwent a right PAO. Measurement d_1_ represents the difference in femoral head height in millimeters. The white line represents the LCEA to the sourcil edge based on the ischia compared with the LCEA to the sourcil edge based on the ground (black line). (LCEA, lateral center‐edge angle; PAO, periacetabular osteotomy.)

**FIGURE 2:**
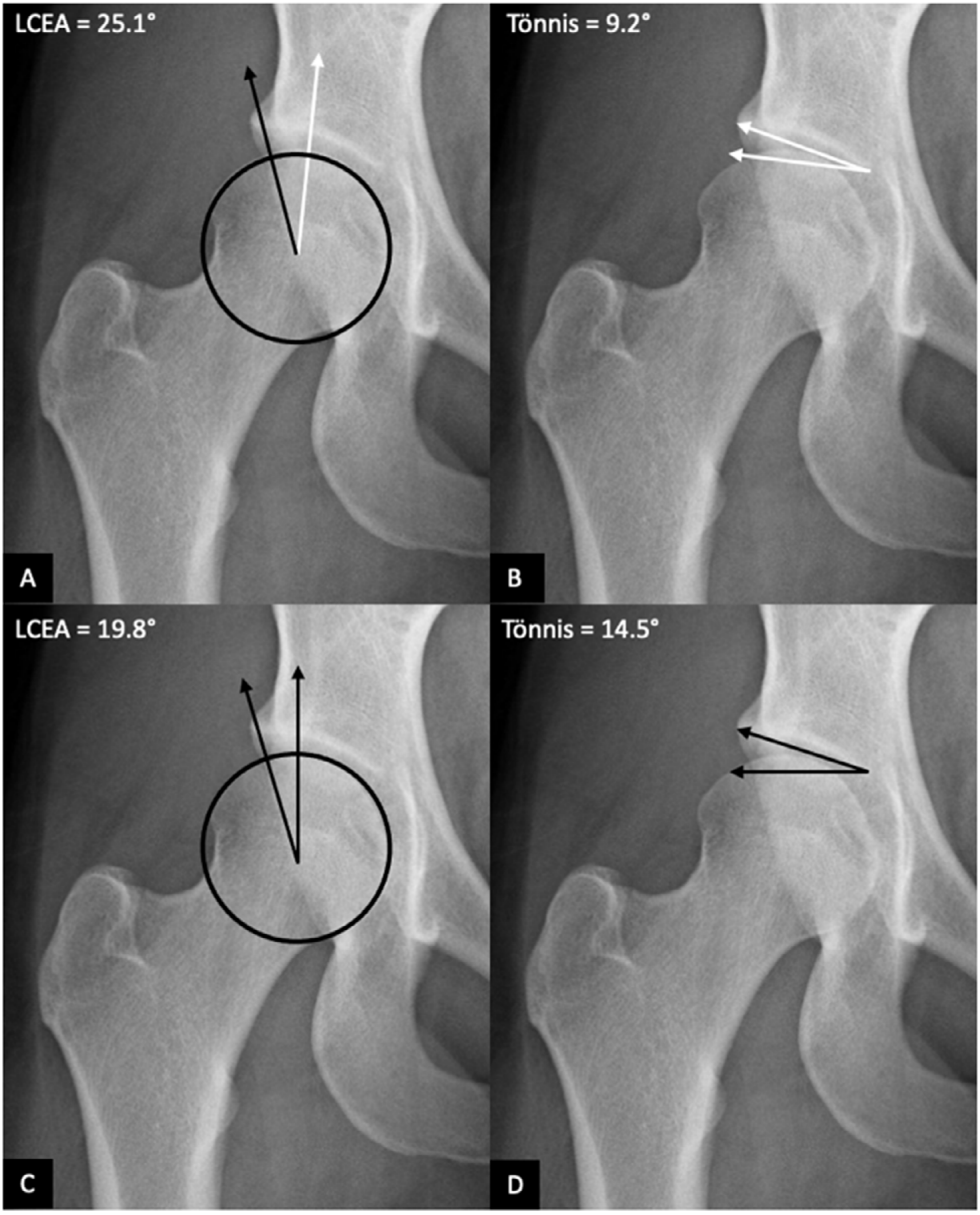
Close‐up of the anteroposterior radiograph of the right hip from Figure 1 with the (A) LCEA and (B) Tönnis angle based on the pelvis in white compared with (C) the LCEA and (D) Tönnis angle based on the ground in black. Pelvic‐based LCEA and Tönnis angle overestimate and underestimate the coverage of the femoral head by approximately 5.3° compared with a ground‐based LCEA in this patient. (LCEA, lateral center‐edge angle.)

### Statistical Analysis

All statistical analyses were performed in SPSS v29.0 (IBM). Differences in degrees between the pelvic‐based and ground‐based measurements were calculated. Univariate linear regression analysis was then performed using the difference in femoral height in millimeters as the independent variable and the difference in degrees between pelvic‐ and ground‐based LCEA or TA measurements for each patient as the dependent variable. Intraclass correlation coefficients (ICCs) were calculated between radiographic reviewers (D.C.L., B.T.J.). Each reviewer independently measured a separate subset of patients, with an overlapping cohort of 15 patients measured by both reviewers to calculate the interobserver reliability. The sample of 15 patients for interobserver ICC calculation was determined based on published guidelines recommending a minimum of 13 to 15 patients for 2‐rater reliability studies with an expected ICC > 0.90.^21^ Interobserver ICCs were calculated between radiographic reviewers using a 2‐way mixed‐effects model with absolute agreement. The significance was set to a *P* value <.05.

## RESULTS

In total, 50 hip arthroscopy patients (100 hips) were included, 32 (64%) were women, the mean age was 34.5 ± 12.4 years, and the mean body mass index was 26.5 ± 5.61 (Figure 3 and Table 1). ICCs were 0.945 (0.727‐0.989) for LCEA (sourcil), 0.935 (0.675‐0.987) for LCEA (bone/lateral most rim), and 0.928 (0.638‐0.985) for TAs, indicating excellent agreement. The mean femoral head height difference in the hip arthroscopy patients was 4.9 ± 3.68 mm. Overall, 30 (60%) hip arthroscopy patients were more symptomatic on the hip associated with the longer limp/elevated hip. This association increased to 65% (11/17) when the femoral head height difference was ≥5 mm and 75% (6/8) when there was a ≥10 mm discrepancy (Figure 4).

**FIGURE 3:**
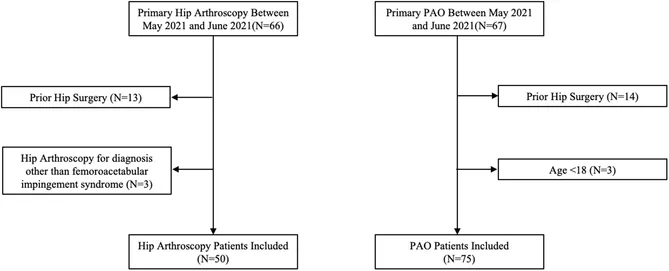
Flow diagram showing the inclusion/exclusion criteria of primary hip arthroscopy and primary PAO patients. (PAO, periacetabular osteotomy.)

**TABLE 1:** Patient Demographics and Rate of the More Elevated Hip Being More Symptomatic Compared With the Lower Hip by Amount of Femoral Head Height Difference

	**Hip Arthroscopy (n = 50)**	**PAO (n = 75)**	**Combined (n = 125)**
	**N (%), Mean (SD)**	**N (%), Mean (SD)**	**N (%), Mean (SD)**
Female sex	32 (64%)	72 (96%)	104 (83%)
Age, yr	34.5 ± 12.4 (18.1‐63.4)	25.2 ± 5.21 (19.0‐36.4)	28.9 ± 9.9 (18.1‐63.4)
BMI	26.5 ± 5.61 (16.5‐41.8)	24.3 ± 3.62 (16.4‐34.2)	25.1 ± 4.6 (16.4‐41.8)
Femoral head difference, mm	4.90 ± 3.68 (0.8‐18.9)	4.98 ± 3.79 (0.4‐18.2)	4.95 ± 3.73 (0.4‐18.9)
Overall elevated hip being more symptomatic	30/50 (60%)	46/75 (61%)	76/125 (61%)
<5 mm difference with elevated side being more symptomatic	13/25 (52%)	20/36 (56%)	33/61 (54%)
≥5 mm difference with elevated being more symptomatic	11/17 (65%)	18/31(58%)	29/48 (60%)
≥10 mm difference with elevated side being more symptomatic	6/8 (75%)	8/8 (100%)	14/16 (88%)
Female sex with more elevated hip being more symptomatic	20/32 (63%)	44/72 (61%)	64/104 (62%)

*Note*: Values represented as either N/total (%) or mean ± SD.

BMI, body mass index; mm, millimeters; PAO, periacetabular osteotomy; SD, standard deviation; yr, years.

**FIGURE 4:**
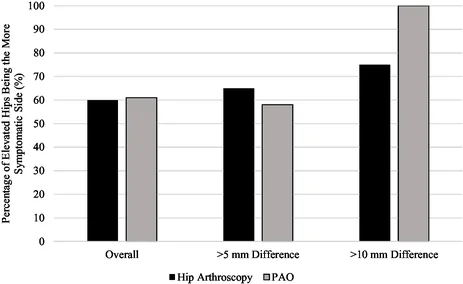
Graphic display of the percentage of patients who reported the more elevated side as being the more painful or unstable side. (PAO, periacetabular osteotomy.)

The 75 patients who underwent PAO were predominantly women (96%, 72/75) and had a mean age of 25.2 ± 5.21 years and a mean body mass index of 24.3 ± 3.62 points. The mean femoral head height difference in PAO patients was 4.98 ± 3.79 mm. Overall, the more symptomatic hip was on the elevated side in 61% (46/75) of PAO patients and 58% (18/31) when there was a ≥5 mm discrepancy and further increased to 100% (8/8) of patients with a ≥10 mm discrepancy (Figure 4).

For patients undergoing primary hip arthroscopy (n = 100) and PAO (n = 75), regression analysis predicting the difference in measurement angle between standard pelvic‐based measurements and ground‐based measurements was performed to determine the amount of angle change that occurs for every 1 mm of femoral head height difference. When compared with the standard pelvic‐based measurement, ground‐based measurements of LCEA to the sourcil edge (*B* = 0.298; *R* ^2^ = 0.775; 95% confidence interval = 0.274‐0.322; *P* < .001) and LCEA to the bone edge (*B* = 0.287; *R* ^2^ = 0.777; 95% confidence interval = 0.264‐0.310; *P* < .001) tended to decrease significantly, whereas TA increased significantly (*B* = 0.296; *R* ^2^ = 0.758; 95% confidence interval = 0.271‐0.321; *P* < .001) when looking at the elevated hip on AP radiograph (Table 2). Although absolute differences were used to ensure that upper and lower hip measurement differences did not counteract each other when comparing ground‐ and pelvic‐based measurements, the relation stated above would simply be inverted when examining the nonelevated hip.

**TABLE 2:** Mean, SD, and Range of LCEA and Measurements and Tönnis Angle Measurements for Hip Arthroscopy Patients, PAO Patients, and the Combined Cohort

**Measurement**	**Hip Arthroscopy (n = 100)**	**PAO (n = 75)**	**Overall (n = 175)**	**Regression Coefficient (** ***B*** **) (95% CI);** ***P*** **Value**
LCEA (sourcil), °				
Pelvic‐based	29.7 ± 6.6 (17.5‐42.4)	16.7 ± 5.5 (3.0‐24.8)	24.1 ± 8.9 (3.0‐42.4)	‐
Ground‐based	29.7 ± 6.7 (16.6‐43.8)	16.2 ± 5.5 (3.2‐24.9)	23.9 ± 9.1 (3.2‐43.8)	‐
Difference (|pelvic − ground|)	2.0 ± 1.3 (0.3‐6.0)	1.9 ± 1.2 (0.1‐6.4)	2.0 ± 1.3 (0.1‐6.4)	*B* = 0.298 (0.274‐0.322) *P* < .001
LCEA (bone), °				
Pelvic‐based	34.7 ± 7.3 (21.8‐53.3)	22.2 ± 5.2 (7.9‐26.4)	29.3 ± 9.0 (7.9‐53.3)	‐
Ground‐based	34.7 ± 7.4 (21.3‐55.7)	21.8 ± 5.1 (8.1‐26.9)	29.2 ± 9.1 (8.1‐55.7)	‐
Difference (|pelvic − ground|)	2.0 ± 1.3 (0.3‐6.3)	1.8 ± 1.1 (0.1‐6.0)	1.9 ± 1.2 (0.1‐6.3)	*B* = 0.287 (0.264‐0.310) *P* < .001
Tönnis angle, °				
Pelvic‐based	3.8 ± 4.4 (−6.0 to 12.6)	13.0 ± 4.2 (2.0‐26.2)	7.7 ± 6.3 (−6.0 to 26.2)	‐
Ground‐based	3.6 ± 4.6 (−9.7 to 13.2)	13.5 ± 4.2 (4.4‐23.3)	7.8 ± 6.6 (−9.7 to 23.3)	‐
Difference (|ground − pelvic|)	1.9 ± 1.3 (0.3‐6.2)	1.8 ± 1.2 (0.1‐6.3)	1.9 ± 1.3 (0.1‐6.3)	*B* = 0.296 (0.271‐0.321) *P* < .001

*Note*: Regression analysis outputs for the differences in pelvic‐ and ground‐based measurements and differences in femoral head height are depicted in the far‐right column.

CI, confidence interval; LCEA, lateral center‐edge angle; PAO, periacetabular osteotomy; SD, standard deviation.

## DISCUSSION

This study revealed that for every 1 mm of difference in femoral head height, measurements change by approximately 0.3° of either over‐ or undercoverage. Furthermore, when comparing the femoral head height on standing AP radiographs, the elevated side was more often symptomatic. Given that the symptomatic hip was more commonly the elevated hip and pelvic‐based measurements produce larger LCEA to the sourcil edge compared with the ground‐based measurements in the elevated hip, adding a ground‐based measurement to the radiographic assessment may shed additional light on this concept of functional acetabular coverage.

Park et al. also sought to investigate the impact of LLD on ground‐ versus pelvic‐based LCEA measurements in pediatric patients with a diagnosed LLD.^14^ According to their search, approximately 188/229 (82%) studies did not specify a pelvic‐ or ground‐based reference for LCEA.^14^ They created a trigonometric equation with the width of the pelvis and LLD to predict differences in measured LCEA based on the pelvis compared with ground‐based measurements. This equation was found to correlate well with changes in ground‐ versus pelvic‐based measurements and could be used to correct for this difference. However, they did not correlate these findings with the effect of femoral head height discrepancies on side‐specific symptoms, nor did they evaluate the effect of leg length on TA measurements.

Although the use of LCEA is common among hip surgeons, multiple studies have addressed its shortcomings in determining true acetabular coverage.^13^ Domb et al. showed that LCEA was underestimated on plain radiographs compared with 3‐dimensional computed tomography reconstructions.^13^ Furthermore, when comparing different radiographic horizons, they found that a ground‐based measurement was significantly different from an ischial‐based measurement, although they did not specify over‐ or undercoverage.^13^ Although we did not compare radiographs to 3‐dimensional computed tomography reconstructions, plain radiographs remain the standard imaging modality used by hip surgeons for pre‐ and postoperative acetabular coverage comparison. Furthermore, our study also revealed a significant difference between ground‐ and pelvic‐based measurements.

Although traditionally LLDs are said to not be of clinical orthopaedic concern until they reach 10 or more millimeters of difference (prevalence of approximately 15% in the normal population), some argue that any slight differences can cause discomfort.^22^, ^23^ Certainly, on the hip arthroplasty side of hip surgery, this has been true.^24^, ^25^, ^26^ Among all patients in this study, the higher hip tended to be more symptomatic in both the PAO (61%) and hip arthroscopy (60%) patients. Furthermore, in patients with an LLD ≥ 10 mm, 100% of PAO patients (8/8) and 75% (6/8) of primary hip arthroscopy patients were more symptomatic on the longer extremity/higher hip. Within the orthopaedic literature, there also tends to be agreement that the longer limb incurs more hip pathology compared with the shorter limb.^27^, ^28^, ^29^ Such pathology in the elevated hip includes greater pain, higher rates of osteoarthritis, and greater instability.^25^, ^27^, ^28^, ^29^ This data highlights that a ground‐based LCEA and TA may be helpful in determining a functional coverage measurement. Future studies are needed to determine if ground‐based or pelvic‐based measurements correlate with overall patient outcomes and patient symptomology after hip arthroscopy and PAOs.

Functional ground‐based measurements may be of greater importance to patients on the ends of the acetabular coverage spectrum. In looking at dysplastic hips, their functional coverage was significantly smaller in their elevated hip compared with the standard pelvic‐based measurement and was more frequently associated with more severe symptoms. This is important regarding increasing severity of acetabular dysplasia because prior research has linked the magnitude of LCEA to the progression of osteoarthritis with every 1° lost below 28° increasing the likelihood by 13%.^30^, ^31^

### Limitations

This study is not without limitations. First, this study used the ischial tuberosities as the pelvic‐based reference line. Using different pelvic‐based reference lines such as teardrop or femoral head horizon lines may impact the specific degree per height difference change seen in the current study; however, prior studies have shown minimal differences between these measures.^7^, ^28^ Second, this study used differences in hip center height, a measure of pelvic obliquity, as a proxy for LLD on a standard AP pelvis radiograph, not full‐length bilateral lower‐extremity standing radiographs. There may be other reasons for pelvic obliquity other than LLD (scoliosis, pelvic tilt/rotation, etc.). Third, a correlation of worse symptoms with the higher hip/longer leg and associating these symptoms with an LLD as the cause of these symptoms may be circular reasoning and a false correlation. Fourth, an a priori power analysis was not performed as this study used all eligible patients during a specific time period with limited patients in the highest categories of femoral head discrepancies. Lastly, this study is a single‐institution study and only included surgical patients, limiting generalizability and inducing selection bias.

## CONCLUSIONS

Differences in femoral head height on standing AP radiographs impact ground‐ versus pelvic‐based radiographic measurements of acetabular coverage by approximately 0.3° per millimeter of femoral head height difference and may result in functional undercoverage of the elevated hip.

## DISCLOSURES

The authors (C.L.P., L.A.A., S.K.A.) declare the following financial interests/personal relationships which may be considered as potential competing interests: C.L.P. receives royalties, is a paid consultant, and receives research support from Zimmer Biomet; is an unpaid consultant for OrthoGrid; owns stock or stock options in OrthoGrid and CoNextions; and is a board member and has committee appointments with the Knee Society. L.A.A. declares relationships with Medacta USA and OrthoGrid Systems: type: other professional activities and SICOT: hip and knee arthroplasty section editor: type: editorial or governing board (self). S.K.A. reports a relationship with Stryker Orthopaedics that includes: consulting or advisory. The other authors (B.T.J., D.C.L., A.K.M., A.L.M.) declare that they have no known competing financial interests or personal relationships that could have appeared to influence the work reported in this article.
